# Exploring Factors Influencing the Physical and Mental Quality of Life of Caregivers of Cancer Patients: A Cross-Sectional Study at Hassan II University Hospital Center

**DOI:** 10.7759/cureus.70074

**Published:** 2024-09-24

**Authors:** Hiba Bourissi, Hind Bourkhime, Karima El Rhazi, Soufiane Mellas

**Affiliations:** 1 Laboratory of Anatomy, Surgery, and Anesthesiology, Faculty of Medicine, Pharmacy, and Dental Medicine, Sidi Mohamed Ben Abdellah University, Fez, MAR; 2 Laboratory of Epidemiology, Clinical Research, and Community Health, Faculty of Medicine and Pharmacy, Sidi Mohamed Ben Abdellah University, Fez, MAR; 3 Laboratory of Epidemiology and Research in Health Sciences, Faculty of Medicine and Pharmacy, Sidi Mohamed Ben Abdellah University, Fez, MAR

**Keywords:** cancer, family caregiver, mental quality of life, physical quality of life, socio-demographic factors

## Abstract

Background

Cancer is a serious public health problem worldwide, causing an impact not only on the patient's quality of life (QoL) but also on their caregiver. This study aims to assess the socio-demographic characteristics, physical and mental QoL, and associated factors among caregivers of cancer patients at the Hassan II University Hospital Center in Fez, Morocco.

Methods

This is a cross-sectional study of 247 caregivers of cancer patients. Their QoL including physical and mental health aspects was assessed using a 12-item Short Form health survey (SF-12). Statistical analysis was conducted utilizing IBM SPSS Statistics for Windows v26.0 (IBM Corp., Armonk, USA). This included calculating frequency distributions and percentages. The general and socio-demographic data associated with QoL were analyzed using the student's t-test and one-way analysis of variance (ANOVA). Only variables with p-value <0.05 were considered statistically significant.

Results

The majority of the participants were 40 years old and over, and 54.3% of the total participants were women. The average physical QoL score was 47.32 (±7.08), while the average mental QoL score was 31.64 (±14.44). For caregivers, certain personal attributes and circumstances were found to influence their mental and physical QoL. Age, gender, and being unemployed were common factors impacting both the mental and physical well-being of caregivers. Moreover, being married (P=0.019) was an additional factor affecting their physical QoL. In contrast, specific aspects related to the caregiving role itself also played a significant role. Caregivers who were close family members of patients (P=0.0001) and those providing care for patients with genitourinary cancer (P=0.048) experienced a notable detrimental effect on their mental QoL.

Conclusion

This study highlights several factors associated with the poor QoL of caregivers, such as gender, age, professional activity, and the relationship with the patient. It is imperative to establish a specific support system for caregivers of cancer patients, including social and economic assistance, to enhance their physical and mental QoL.

## Introduction

The number of cancer patients being cared for at home is increasing [1]. Consequently, the family plays a crucial role in this home care and maintenance, ensuring both continuity of care and social support [2].

Families, or more specifically, family caregivers (FCGs), are those who assist a relative who is ill, dependent, and requires various care and interventions [3]. There are also primary FCGs, chosen by the patient as the most involved in their care [2].

Caring for a sick relative is not easy; it requires emotional and material commitment and significant responsibility. This can have physical, psychological, and financial consequences, potentially deteriorating the FCGs' quality of life (QoL) [4]. As FCGs play a multifunctional role, requiring much effort, patience, commitment, and strength, significant physical and mental exhaustion is generally associated [5]. FCGs, in turn, need support to adapt to this situation and overcome the stressful events they experience daily [6]. Although the duration of care provided by FCGs of cancer patients is on average shorter than that for FCGs of non-cancer patients (1.9 years vs. 4.1 years, respectively), they endure a much heavier weekly workload (32.9 hours per week vs. 23.9 hours for those caring for non-cancer patients) and intensive daily tasks [7].

The impact of cancer on FCGs has been documented in numerous studies, revealing aspects such as emotional distress, fear, guilt, and concerns [2]. Physically, problems such as digestive disorders like indigestion and changes in appetite, headaches, chronic fatigue, weight changes, and muscle pain have been observed [8]. Moreover, psychological issues such as stress, depression, anxiety, social isolation, concentration difficulties, and exhaustion have been reported [8]. Interestingly, the psychological impact on FCGs can sometimes be more significant than on the patients themselves [2].

The National Plan for the Prevention and Control of Cancer (PNPCC) 2020-2029, endorsed by [9], aims to enhance the well-being of cancer patients and their FCGs in Morocco. Integral to this plan is the third sustainable development goal (SDG 3), which is committed to ensuring health and well-being at all ages [10].

Our study contributes to the PNPCC's broader goals by focusing on the QoL of cancer patients' FCGs, assessing their physical and mental well-being and identifying contributing factors.

## Materials and methods

Target population and data collection

This cross-sectional study was conducted from April 2023 to July 2023 at the oncology department of Hassan II University Hospital Center (CHU) in Fez, Morocco, which acts as a regional oncology center.

Our target population consisted of Moroccan adults aged 18 and over who were the primary caregivers of cancer patients, excluding those who were themselves cancer patients or who were suffering from memory disorders at the time of data collection.

We recruited 247 individuals from the waiting rooms during their patients' chemotherapy sessions as well as during visits to inpatients at the oncology department. The FCGs completed a psychometrically validated questionnaire, which was translated and adapted to the Moroccan context. On average, our survey took about six minutes.

Authorizations and questionnaire

The research protocol was evaluated and approved by the Faculty of Medicine and Pharmacy Ethics Committee at the Sidi Mohamed Ben Abdellah University in Fez under approval number 2/22. 

Authorization to conduct the study at the oncology service was granted, and informed consent was obtained from each participant. The study was conducted concerning their rights and ethical considerations.

General and socio-demographic data were collected, including age, gender, region, marital status, occupation, relationship to the patient, location of cancer, etc. (see Appendices).

The QoL of FCGs of cancer patients was comprehensively assessed using a 12-item Short Form health survey (SF-12) [11]. The SF-12, a condensed iteration of the more extensive 36-item Short Form health survey (SF-36), is designed to efficiently gauge various dimensions of health and well-being while minimizing respondent burden. It comprises 12 questions that cumulatively represent eight scaled scores, each focusing on a distinct aspect of health and functioning. These eight scales include vitality, physical functioning, bodily pain, general health perceptions, role-physical functioning, role-emotional functioning, social role functioning, and mental health, highlighting both physical and mental aspects of QoL (see Appendices).

Ethical considerations

All participants were informed in writing and orally about the purpose of our study. Anonymity, confidentiality, and privacy were assured. Our questionnaire was administered in a quiet, separate area that guaranteed their privacy; no third party who could influence the response was present. The questionnaires were kept confidential by the project team, and the database containing the participants' responses was protected, managed, and processed confidentially. Participants' rights to freely choose to participate in our study and to withdraw at any time were ensured.

Data processing and analysis

The data were entered into Microsoft Excel (Microsoft Corp., Redmond, USA) and then transferred to IBM SPSS Statistics for Windows v26.0 (IBM Corp., Armonk, USA) for potential analyses. Frequency distributions and percentages were calculated for general and socio-demographic variables (qualitative variables). Mean values with standard deviations were calculated for QoL (quantitative variable).

The independent variables of this study were the general and socio-demographic characteristics, while the dependent variable was the QoL of the FCGs. The general and socio-demographic data associated with QoL were analyzed using the student's t-test and one-way analysis of variance (ANOVA). Only variables with p-value <0.05 were considered statistically significant.

## Results

Socio-demographic characteristics and QoL of participants

We surveyed 247 FCGs of cancer patients, of whom 208 responded to the questionnaire, representing a response rate of 84.21%. All participants were aged 18 and over, with an average age of 37.04±11.47 years. The majority were 40 years or older (n=123, 59.1%), female (n=113, 54.3%), married (n=138, 66.3%), close family members of the patient (n=190, 91.3%), not engaged in professional activity (n=138, 66.3%), residing in the Fez-Meknès region (n=178, 85.6%), caring for patients with genitourinary cancer (n=109, 52.4%), and spending less than 300 MAD to reach the care facility (n=141, 67.8%) (Table 1). Regarding the duration of caregiving, 66 caregivers (n=66, 31.7%) had been providing care for less than six months, 99 caregivers (n=99, 47.6%) for six months to a year, and 43 caregivers (n=43, 20.7%) for more than a year.

**Table 1:** Descriptive demographic characteristics and their impact on physical and mental health outcomes of cancer patients' FCGs ₁ Widowed, single, divorced; ₂ Student, unemployed, retired; ₃ Pulmonary, digestive, cerebral, ENT (ear, nose, throat), lymphomas, osteosarcomas; ₄ Beni Mellal-Khenifra, Drâa-Tafilalet, Oriental, Tanger-Tetouan-Al Hoceïma; SF-12: 12-item Short Form health survey; FCGs: Family caregivers

Socio-demographic parameters	N (percentage)	SF-12 physical	SF-12 mental
		Mean±SD	Mean±SD
Age			
18-39	85 (40.9%)	50.84±5.27	35.13±14.18
40 and older	123 (59.1%)	44.89±7.17	29.23±14.17
P-value		P=0.0001	P=0.004
Gender			
Male	95 (45.7%)	48.47±6.50	36.30±13.53
Female	113 (54.3%)	46.36±7.42	27.72±14.05
P-value		P=0.032	P=0.0001
Marital status			
Not married ₁	70 (33.7%)	48.94±7.29	31.78±14.76
Married	138 (66.3%)	46.51±6.85	31.57±14.32
P-value		P=0.019	P=0.919
Professional activity			
In professional activity	70 (33.7%)	50.13±5.41	37.53±13.88
Non-working ₂	138 (66.3%)	45.95±7.41	28.65±13.83
P-value		P=0.0001	P=0.0001
Relationship with the patient			
Close family (father, mother, brother, sister, husband or wife)	190 (91.3%)	47.21±7.28	30.39±13.68
Extended family/neighbors (aunt, uncle, grandparents, etc.)	18 (8.7%)	48.55± 4.42	44.86±15.96
P-value		P=0.443	P=0.0001
Location of cancer			
Genitourinary cancers	109 (52.4%)	47.52±7.37	33.52±14.55
Other cancers ₃	99 (47.6%)	47.10±6.78	29.57±14.09
P-value		P=0.668	P=0.048
Region			
Fez-Meknès	178 (85.6%)	47.44±7.12	30.73±14.26
Other region ₄	30 (14.4%)	46.66±6.93	37.01±14.55
P-value		P=0.579	P=0.027
				Transportation costs			
Less than 300 Dh	141 (67.8%)	47.78±7.01	31.84±13.91
More than 300 Dh	67 (32.2%)	46.37±7.18	31.22±15.58
P-value		P=0.182	P=0.775
Duration of caregiving			
Less than six mounth	66 (31.7%)	46.94±6.47	33.77±15.28
Six months to a year	99 (47.6%)	47.85±7.22	31.69±13.27
More than one year	43 (20.7%)	46.70±7.69	28.26±15.37
P-value		P=0.583	P=0.150

The average physical QoL score was 47.32 (±7.08), while the average mental QoL score was 31.64 (±14.44) (Table 2). 

**Table 2:** Average scores for physical and mental QoL QoL: Quality of life

QoL aspect	Average score	Standard deviation
Physical QoL	47.32	7.08
Mental QoL	31.64	14.44

The physical and mental QoL scores were derived using the SF-12 instrument following the method developed by the creators of the questionnaire. Each response was converted into a score and then these scores were combined using standard methods to get two summary scores: one representing physical health (PCS), and the other reflecting mental health (MCS). These scores were adjusted to have an average of 50 and a standard deviation of 10 for easier comparison.

Factors associated with the QoL of caregivers

Physical QoL

We found that being aged 40 and over (P=0.0001), female gender (P=0.032), married (P=0.019), and not having a professional activity (P=0.0001) were all factors negatively associated with the physical QoL (SF12) of FCGs of cancer patients (Table 1).

Mental QoL

Similarly, we found that being 40 years or older (P=0.004), female (P=0.0001), not engaged in professional activity (P=0.0001), having close family ties to the patient (such as being a parent, spouse, or sibling) (P=0.0001), and caring for a patient with genitourinary cancer (P=0.048) were all significantly and negatively associated with the FCGs' QoL (Table 1). In addition, regional differences indicated that caregivers outside of the Fez-Meknès region reported better mental health (P=0.027) (Table 1).

Correlation

The analysis revealed a significantly positive association between mental QoL and physical QoL, although this relationship remained weak (r=0.272; P=0.0001) (Table 3).

**Table 3:** Correlation between mental and physical QoL scores QoL: Quality of life

Relationship evaluated	Correlation coefficient (r)	Significance level (P-value)	Interpretation
Association between mental and physical QoL	0.272	<0.0001	A significant but weak positive link exists between mental and physical QoL, suggesting improvements in one may slightly enhance the other.

## Discussion

To the best of our knowledge, this research is the first in Morocco to explore the QoL parameters of FCGs of cancer patients. It underscores our aim to assess the physical and mental well-being of these caregivers, along with identifying the diverse factors that influence it.

The present study revealed that most participants were over 40, and women constituted 54.3% of this group. The average scores for physical and mental QoL stood at 47.32 and 31.64, respectively. Influential factors for the well-being of FCGs included gender, age over 40, marital status, and employment status. Furthermore, the caregiving role, notably for close family members and patients with genitourinary cancer, had a significant impact on their mental health.

The findings of our study are in line with other similar research, highlighting that the majority of FCGs are women, married, and close family members of the patient [12,13], and typically spouses or parents [14,15]. In our Moroccan context, these observations are linked to socio-cultural and religious factors. However, this family solidarity can come at a high cost. The FCGs, who must make time for their sick relative, may have to leave their job depending on their professional responsibilities. Since the role of the FCGs is not officially recognized in Morocco, they are left without rights, and therefore, without employment. In this regard, our results reveal that the majority of FCGs were not engaged in professional activity, similar to their counterparts in Turkey where the majority (88.1%) were unemployed [12]. Kilic et al. also reported that nearly half of the FCGs of cancer patients (48.4%) were out of work [8].

Despite the challenges, FCGs still play a very important role in the care of cancer patients but at the expense of their own QoL. This study revealed that the QoL of FCGs of cancer patients was very low, both physically and mentally. Several studies conducted with similar populations have shown that their QoL was poor, with the most affected dimensions being those related to the mental and emotional health of the caregiver [16,17].

Additionally, more unfavorable QoL is observed in women compared to men due to several socio-cultural factors, such as the traditional role of Moroccan women within the family, societal expectations, less access to necessary support resources, etc. This observation highlights the role that women play in the majority of societies. Our results support the findings of Kilic et al. who demonstrated a significant impact between female gender and the QoL of FCGs [8] and those of Shin et al. who emphasized that women had a deteriorated QoL [18].

It is also interesting to note that being 40 years old and over was significantly associated with a less favorable physical and mental QoL, which contradicts the findings of Mellon et al. who concluded that being older was rather in favor of a more appreciated QoL [19]. In our case, this could be linked to several factors, such as the increasing responsibilities with age including financial constraints, personal or professional exhaustion, or social isolation. Marital status influences the physical QoL of FCGs, while the family relationship with the patient affects their mental QoL. Our findings are in line with the work of [20,21], which concluded that marital status and the relationship with the patient significantly affect the FCGs' QoL.

The unemployed FCGs experienced deterioration in both their physical and mental QoL, possibly linked to significant financial constraints due to the lack of income, which generates feelings of uselessness and devaluation in the FCGs. In line with this, Kilic et al. reported statistically significant differences in terms of unemployment and the QoL of FCGs [8]. Unlike our study where FCGs of patients with genitourinary cancer showed a significant deterioration in their mental QoL, Kilic et al. did not observe any significant difference between the types of cancer of the patients and the QoL scores of the FCGs [8]. However, Hyde and colleagues highlighted a higher burden among caregivers of patients with prostate cancer, which was found to be related to distress, anxiety, depression, and cancer-specific distress over time [22]. It is important to note that any type of cancer could affect the QoL of FCGs. However, genitourinary cancers generally require invasive treatments and are likely to lead to postoperative complications and affect the patient's intimate sphere. This could cause more stress and discomfort for the FCGs, especially when it comes to discussing subjects considered intimate, given that sexuality remains a taboo subject in Morocco. Genitourinary cancers can cause psychosocial concerns for FCGs, which could impact their QoL.

While this study has certain limitations, such as being conducted exclusively at the University Hospital Center in Fez, it offers valuable insights that could inform future research in various healthcare environments. Although we were unable to directly assist FCGs of cancer patients identified as having poor QoL, we took proactive measures by encouraging them to seek professional help in psychology and mental health, which could significantly enhance their well-being.

## Conclusions

The QoL of FCGs of cancer patients was significantly low. This decline was influenced by several factors, particularly gender, age, and professional activity in terms of physical and mental aspects. The findings of our study are particularly relevant for healthcare professionals in oncology services, especially nurses. Our outcomes indicate that it is crucial to shift focus toward FCGs, to understand their challenges and needs, and to provide them with targeted interventions. In light of these findings, it is imperative to design suitable and tailored interventions aimed at improving the QoL for FCGs of cancer patients. Nurses can be active members in this process given their clinical expertise and skills in therapeutic education. Furthermore, our study supports the guidelines of the Lalla Salma Cancer Plan, which emphasizes the importance of improving the QoL for FCGs of cancer patients. Therefore, it is recommended that future studies continue in this direction.

## Appendices

I. Socio-demographic questionnaire

We used a socio-demographic questionnaire to collect information on variables such as age, gender, marital status, occupation, etc. This allowed us to gather a comprehensive profile of the participants and better understand the factors that may influence their quality of life. The questionnaire was as follows:

1. Age: 18-39 years old 40-59 years old 60 years or older

2. Gender: Male Female

3. City: ..................................................................

4. Marital Status: Single Married Divorced Widowed

5. Occupation (where do you work?): ..................................................................

6. What is your relationship to the patient? My brother, sister My mother, father My husband, wife My friend (male or female) My neighbor (male or female) None of the above: (please specify your relationship) ..................................................................

7. Number of trips to the university hospital center per month: Once a month Twice a month More than twice a month

8. What type of cancer does the patient have (where is the cancer located in their body)? ..................................................................

9. How long have you been caring for the patient? Less than six months Between six months and one year More than one year

10. How much does it cost you to travel (round trip) to Hassan II University Hospital Center? Less than 100 dirhams Between 100 dirhams and 300 dirhams Between 300 dirhams and 500 dirhams More than 500 dirhams

II. 12-item Short Form health survey (SF-12)

SF-12 is a measure of health status, often used as a measure of quality of life. It is the shortened version of the SF-36 and consists of eight scaled scores. These include vitality, physical functioning, bodily pain, general health perceptions, physical role functioning, emotional role functioning, social role functioning, and mental health. The SF-12 was as follows:

This survey asks for your views about your health. This information will help keep track of how you feel and how well you are able to do your usual activities. Answer each question by choosing just one answer. If you are unsure how to answer a question, please give the best answer you can.

In general, would you say your health is: 1. Excellent 2. Very good 3. Good 4. Fair 5. Poor

The following questions are about activities you might do during a typical day. Does your health now limit you in these activities? If so, how much?

Moderate activities such as moving a table, pushing a vacuum cleaner, bowling, or playing golf. 1. Yes, limited a lot 2. Yes, limited a little 3. No, not limited at all

Climbing several flights of stairs. 1. Yes, limited a lot 2. Yes, limited a little 3. No, not limited at all

During the past four weeks, have you had any of the following problems with your work or other regular daily activities as a result of your physical health?

Accomplished less than you would like. 1. Yes 2. No

Were limited in the kind of work or other activities. 1. Yes 2. No

During the past four weeks, have you had any of the following problems with your work or other regular daily activities as a result of any emotional problems (such as feeling depressed or anxious)?

Accomplished less than you would like. 1. Yes 2. No

Did work or activities less carefully than usual. 1. Yes 2. No

During the past four weeks, how much did pain interfere with your normal work (including work outside the home and housework)? 1. Not at all 2. A little bit 3. Moderately 4. Quite a bit 5. Extremely

These questions are about how you have been feeling during the past four weeks. For each question, please give the one answer that comes closest to the way you have been feeling.

Have you felt calm and peaceful? 1. All of the time 2. Most of the time 3. A good bit of the time 4. Some of the time 5. A little of the time 6. None of the time

Did you have a lot of energy? 1. All of the time 2. Most of the time 3. A good bit of the time 4. Some of the time 5. A little of the time 6. None of the time

Have you felt downhearted and blue? 1. All of the time 2. Most of the time 3. A good bit of the time 4. Some of the time 5. A little of the time 6. None of the time

During the past four weeks, how much of the time has your physical health or emotional problems interfered with your social activities (like visiting friends, relatives, etc.)? 1. All of the time 2. Most of the time 3. Some of the time 4. A little of the time 5. None of the time
